# Impact of Prior Angiotensin‐Converting Enzyme Inhibitor and Angiotensin II Receptor Blocker Use on Delirium Incidence in ICU Patients: A Retrospective Study

**DOI:** 10.1002/hsr2.72676

**Published:** 2026-06-22

**Authors:** Zhe Li, Shuo Liu, Qihai Wan, Yahui Niu, Yi Yu

**Affiliations:** ^1^ Department of Intensive Care Medicine Juancheng County People's Hospital Heze China; ^2^ Department of Gastroenterology Juancheng County People's Hospital Heze China; ^3^ Department of Anesthesiology, West China Second University Hospital Sichuan University Chengdu China; ^4^ Key Laboratory of Birth Defects and Related Diseases of Women and Children (Sichuan University) Ministry of Education Chengdu China; ^5^ Department of Critical Care Medicine The Second Affiliated Hospital of Guangzhou University of Chinese Medicine Guangzhou P. R. China

**Keywords:** acute kidney injury, angiotensin II receptor blocker, angiotensin‐converting enzyme inhibitor, Delirium, intensive care unit

## Abstract

**Background and Aims:**

This investigation aimed at exploring the impact that the pre‐existing use of angiotensin‐converting enzyme inhibitors (ACEIs) and angiotensin II receptor blockers (ARBs) has on the incidence of delirium in patients within the intensive care unit (ICU).

**Methods:**

A retrospective investigation was conducted by utilizing the Medical Information Mart for Intensive Care dataset spanning from 2008 to 2022 (version 3.0). The primary endpoint of the study was the onset of delirium during the patients' 30‐day stay in the ICU. Secondary endpoints incorporated the duration of ICU stay, the period of vasoactive drug administration, the incidence rate of acute kidney injury (AKI), and the necessity for continuous renal replacement therapy (CRRT).

**Results:**

A total of 15,666 patients were included in the analysis. Among them, 6,942 patients had no prior use of ACEIs or ARBs, 6,643 patients used ACEIs, and 2,081 patients used ARBs. The mean age of the patients was 66.2 ± 15.5 years, 56.4% were male, and 62% experienced delirium. Multivariable Cox regression models revealed significant hazard ratios (HRs) for patients who received ACEIs and ARBs. After adjusting for all confounding variables, both ACEIs and ARBs were significantly associated with a reduced risk of delirium (HR: 0.13, 95% CI: 0.13–0.15 for ACEIs; HR: 0.13, 95% CI: 0.12–0.14 for ARBs). The use of ACEIs was significantly associated with a lower risk of AKI (HR: 0.9, 95% CI: 0.82–0.98). Additionally, both ACEIs and ARBs were significantly associated with a decreased need for CRRT.

**Conclusion:**

Despite the significant association between the utilization of ACEIs or ARBs and a reduced incidence of delirium in critically ill patients, additional research is necessary to validate these results.

**Abbreviations**
ACEIs: Angiotensin‐converting enzyme inhibitors
AKI: Acute kidney injury
ARBs: Angiotensin II receptor blockers
BMI: Body mass index
CAM‐ICU: Confusion Assessment Method for the ICU
CRRT: Continuous renal replacement therapy
HR: Hazard ratio
ICU: Intensive care unit
MIMIC‐IV: Medical Information Mart for Intensive Care
OR: Odds ratio
RAAS: Renin–angiotensin–aldosterone system
SOFA: Sequential Organ Failure Assessment
SpO_2_
: Oxygen saturation
STROBE: Strengthening the Reporting of Observational Studies in Epidemiology
WBC: White blood cell

## 1 Introduction

Delirium, a prevalent sign of organ dysfunction in critically ill adults, significantly elevates the morbidity and mortality rates [1]. Among patients admitted to intensive care units (ICUs), more than half encounter delirium during their hospital admissions [2, 3]. This condition is independently correlated with an increased risk of death [4]. ersistent delirium is a key risk factor for long‐term cognitive impairment following critical illness, potentially leading to severe disability [5, 6]. Moreover, the onset of delirium is associated with longer hospital stays and higher healthcare expenses [7, 8].

The pathophysiology of delirium has not been fully comprehended. Plausible underlying mechanisms encompass inflammation, insufficient cerebral blood flow, neurotransmitter imbalance, and metabolic disorders [9, 10, 11]. Current treatment options include both pharmacological and nonpharmacological interventions, with nonpharmacological strategies integrated with ABCDEF Bundle treatment programs [12]. Pharmacological options include dexmedetomidine, statins, and ketamine; however, no single agent has shown proven effectiveness in the treatment or prevention of delirium [13, 14, 15, 16]. Notably, the guidelines issued by the Society of Critical Care Medicine do not advocate the regular usage of dexmedetomidine, statins, and ketamine for delirium prevention in critically ill adult patients [17].

Preclinical and observational research suggests that angiotensin ‐ converting enzyme inhibitors (ACEIs) and angiotensin receptor blockers (ARBs) may potentially impede the deterioration of memory function among individuals diagnosed with mild ‐ to ‐ moderate Alzheimer's disease [18]. It is hypothesized that their actions within the brain entail the modulation of the renin–angiotensin–aldosterone system (RAAS) activity [19]. Specifically, ACEIs inhibit angiotensin II production, which is thought to dampen inflammatory responses within the RAAS [20], while ARBs may act by stabilizing the blood–brain barrier and modulating neuronal activity within the RAAS by downregulating angiotensin type 1 receptors in cerebral endothelial cells [21]. Considering the impact of the RAAS on microglial activation and oxidative stress, both of which have an effect on learning and memory, this modulation holds particular significance in the prevention of delirium [22]. However, it remains unclear whether these beneficial effects of ACEIs and ARBs can be applied in inpatient settings, particularly for critically ill patients, and this area is largely under ‐ explored.

Consequently, we carried out a retrospective investigation leveraging the Medical Information Mart for Intensive Care (2008–2019) (MIMIC ‐ IV) dataset. The aim of this research was to examine the relationships between the occurrence rates of delirium and the utilization of ACEIs or ARBs.

## 2 Methods

This investigation incorporated critically ill patients sourced from the MIMIC ‐ IV dataset (version 3.0), who either had exposure to ACEIs or ARBs or were unexposed. The MIMIC ‐ IV, a longitudinal, single ‐ center data repository, encompasses data spanning from 2008 to 2019 [23]. One of the authors of this study, Yi Yu, was granted authorization to utilize this database, with the certificate ID being 6477678. This research was conducted in strict accordance with the Guidelines for Strengthening the Reporting of Observational Studies in Epidemiology (STROBE) [24].

### 2.1 Study Population and Data Retrieval

The current study enrolled adult patients aged 18 years or older who were admitted to the ICU. In instances where patients had multiple ICU admissions, only the first admission was considered for analysis. Patients with an ICU stay of less than 24 h were excluded. Delirium was diagnosed according to the criteria of the Confusion Assessment Method for the Intensive Care Unit (CAM ‐ ICU) [25]. Data regarding patients' demographic characteristics, vital signs, laboratory test results, comorbidities, clinical severity scores, and other admission ‐ related information were gathered.

### 2.2 ACEIs and ARBs

The utilization of ACEIs and ARBs was ascertained according to the prescriptions recorded in the MIMIC ‐ IV database before the patients' admission to the ICU. The ACEIs consisted of medications, such as Benazepril, Captopril, Enalapril, Fosinopril, Lisinopril, Quinapril, Moexipril, and others, and the ARBs consisted of Losartan, Valsartan, Irbesartan, Olmesartan, Candesartan, and other drugs.

### 2.3 Covariates

Delirium risk factors among the patients in the ICUs were documented [26, 27]. The covariates under investigation encompassed age, sex, insurance status, body mass index (BMI), vital signs such as respiratory rate, temperature, and oxygen saturation (SpO2), as well as laboratory parameters including white blood cell (WBC) count, hemoglobin level, platelet count, and glucose level. Moreover, this study gathered data on various health‐related metrics, among which the Sequential Organ Failure Assessment (SOFA) scores were included [28]. Comorbid conditions were also taken into account, including cardiovascular disorders, dementia, liver diseases, kidney ailments, malignancies, neurological conditions, and chronic pulmonary diseases. Demographic data pertaining to race and marital status were also collected.

The matching covariates were selected based on the following principles: **Confounders:** Variables having an impact on both the exposure factor and the outcome were incorporated into the analysis. **Temporal priority:** Variables that manifested prior to the exposure factor were given preference, whereas those that altered in response to the exposure factor were excluded from the analysis. **Outcome‐relevant variables:** Variables that may not directly affect the exposure factor but had a significant impact on the outcome were also included. The presence of multicollinearity among these variables was evaluated through the application of the variance inflation factor (VIF). Specifically, when the value of the VIF exceeded 2, it was regarded as a sign that multicollinearity existed within the dataset.

### 2.4 Adjustment for Baseline Imbalances

Despite random allocation to ACEIs/ARBs groups based on clinical indication, significant baseline imbalances were observed between the “No ACEIs/ARBs” group and the combined ACEIs/ARBs groups in several characteristics, including age, heart failure, sepsis, SOFA scores, and in‐hospital mortality (all *p* < 0.001; Table 1). These imbalances represented potential confounders. To rigorously address this issue and estimate the independent association of ACEI/ARB use with delirium, we constructed a series of multivariable regression models with progressive adjustment.

**Table 1:** Baseline characteristics of the participants.

Variable	Total (*N* = 23,738)	No ACEIs/ARBs (*n* = 6,942)	ACEIs (*n* = 12,141)	ARBs (*n* = 3,403)	ACEIs/ARBs (*n* = 1,252)	*p* value
Age, years	66.6 ± 15.0	63.3 ± 17.7	67.4 ± 13.8	70.6 ± 12.1	66.1 ± 14.0	< 0.001
Sex, male, *n* (%)	13,644 (57.5)	3,814 (54.9)	7,421 (61.1)	1,720 (50.5)	689 (55)	< 0.001
BMI, kg/m^2^	29.6 ± 8.1	28.8 ± 8.4	29.9 ± 8.1	30.4 ± 7.8	30.0 ± 7.2	< 0.001
Race, *n* (%)						< 0.001
White	15,976 (67.3)	4,362 (62.8)	8,480 (69.8)	2,293 (67.4)	841 (67.2)	
Other	7,762 (32.7)	2,580 (37.2)	3,661 (30.2)	1,110 (32.6)	411 (32.8)	
Insurance, *n* (%)						< 0.001
Medicaid	1,396 (5.9)	560 (8.1)	654 (5.4)	117 (3.4)	65 (5.2)	
Medicare	11,721 (49.4)	3,133 (45.1)	6,092 (50.2)	1,826 (53.7)	670 (53.5)	
Other^a^	10,621 (44.7)	3,249 (46.8)	5,395 (44.4)	1,460 (42.9)	517 (41.3)	
Marital status, *n* (%)						< 0.001
Married	12,877 (54.2)	3,581 (51.6)	6,659 (54.8)	1,962 (57.7)	675 (53.9)	
Single	5,749 (24.2)	2,009 (28.9)	2,829 (23.3)	605 (17.8)	306 (24.4)	
Divorced	1,780 (7.5)	561 (8.1)	886 (7.3)	253 (7.4)	80 (6.4)	
Widowed	3,332 (14.0)	791 (11.4)	1,767 (14.6)	583 (17.1)	191 (15.3)	
WBC (×10^9^)	12.4 ± 8.4	13.1 ± 10.2	12.1 ± 7.5	12.1 ± 7.8	11.7 ± 5.7	< 0.001
Hb (g/L)	10.8 ± 2.1	10.6 ± 2.1	10.9 ± 2.1	10.6 ± 2.0	10.5 ± 2.0	< 0.001
Respiration rate (bpm)	19.3 ± 3.7	19.8 ± 4.0	19.1 ± 3.6	18.9 ± 3.4	19.2 ± 3.5	< 0.001
SPO_2_ (%)	97.0 ± 2.0	97.0 ± 2.1	96.9 ± 1.9	96.9 ± 1.9	97.0 ± 2.0	0.002
Heart rate (bpm)	84.4 ± 15.6	87.8 ± 16.7	83.4 ± 15.0	81.9 ± 14.5	81.5 ± 15.0	< 0.001
MAP (mmHg)	78.6 ± 11.2	78.1 ± 10.7	79.0 ± 11.4	78.6 ± 11.0	78.7 ± 11.8	< 0.001
LAC (mmol/L)	2.3 ± 1.5	2.5 ± 1.9	2.2 ± 1.3	2.2 ± 1.2	2.4 ± 1.5	< 0.001
BUN, Median (IQR)	20.0 (14.0, 32.0)	19.5 (13.0, 34.0)	19.5 (14.0, 30.0)	20.5 (15.0, 32.0)	24.0 (16.0, 40.0)	< 0.001
Scr, Median (IQR)	1.0 (0.8, 1.5)	1.0 (0.7, 1.6)	1.0 (0.8, 1.4)	1.0 (0.8, 1.5)	1.2 (0.9, 1.9)	< 0.001
Glucose (mmol/L)	142.8 ± 47.2	138.0 ± 47.3	143.3 ± 46.3	147.4 ± 47.1	151.1 ± 52.3	< 0.001
Charlson comorbidity index	5.8 ± 2.7	5.5 ± 3.1	5.9 ± 2.6	6.4 ± 2.6	6.4 ± 2.5	< 0.001
SOFA score	4.9 ± 3.3	6.0 ± 3.8	4.5 ± 3.1	4.4 ± 3.0	4.8 ± 3.1	< 0.001
Myocardial infarct, *n* (%)	4,572 (19.3)	827 (11.9)	2,805 (23.1)	651 (19.1)	289 (23.1)	< 0.001
Congestive heart failure, *n* (%)	7,134 (30.1)	1,418 (20.4)	4,005 (33)	1,122 (33)	589 (47)	< 0.001
Cerebrovascular disease, *n* (%)	4,067 (17.1)	1,229 (17.7)	2,136 (17.6)	532 (15.6)	170 (13.6)	< 0.001
Dementia, *n* (%)	1,029 (4.3)	471 (6.8)	415 (3.4)	115 (3.4)	28 (2.2)	< 0.001
Chronic pulmonary disease, *n* (%)	5,785 (24.4)	1,675 (24.1)	2,926 (24.1)	871 (25.6)	313 (25)	0.291
Renal_disease, *n* (%)	5,082 (21.4)	1,274 (18.4)	2,420 (19.9)	956 (28.1)	432 (34.5)	< 0.001
Severe liver disease, *n* (%)	903 (3.8)	670 (9.7)	182 (1.5)	42 (1.2)	9 (0.7)	< 0.001
Sepsis, *n* (%)	12,816 (54.0)	4,719 (68)	5,959 (49.1)	1,525 (44.8)	613 (49)	< 0.001
Sepsis shock, *n* (%)	2,157 (9.1)	1,160 (16.7)	703 (5.8)	191 (5.6)	103 (8.2)	< 0.001
Diabetes, *n* (%)	8,307 (35.0)	1,570 (22.7)	4,588 (37.7)	1,525 (44.6)	624 (49.8)	< 0.001
dexmedetomidine, *n* (%)	4,194 (17.7)	1,994 (28.7)	1,648 (13.6)	406 (11.9)	146 (11.7)	< 0.001
Midazolam, *n* (%)	4,438 (18.7)	1,864 (26.9)	1,976 (16.3)	416 (12.2)	182 (14.5)	< 0.001
Propofol, *n* (%)	11,024 (46.4)	4,008 (57.7)	5,167 (42.6)	1,342 (39.4)	507 (40.5)	< 0.001
30‐day mortality, *n* (%)	3,382 (14.2)	1,609 (23.2)	1,261 (10.4)	368 (10.8)	144 (11.5)	< 0.001
90‐day mortality, *n* (%)	3,671 (15.5)	1,759 (25.3)	1,365 (11.2)	388 (11.4)	159 (12.7)	< 0.001
AKI in 7 days, *n* (%)	16,463 (69.4)	5,085 (73.2)	8,126 (66.9)	2,350 (69.1)	902 (72)	< 0.001
CRRT, *n* (%)	1,064 (4.5)	605 (8.7)	315 (2.6)	91 (2.7)	53 (4.2)	< 0.001
Vasoactive time, hours	28.2 ± 86.2	48.4 ± 115.5	20.0 ± 66.6	19.0 ± 79.3	20.5 ± 59.4	< 0.001
Delirium, *n* (%)	8,494 (35.78)	5,742 (82.71)	2,015 (16.59)	532 (15.63)	205 (16.37)	< 0.001

*Note: p* value (No ACEI/ARB vs. All Others). For each variable, the mean ± standard deviation, median (interquartile range), or number (percentage) was reported (as appropriate). Categorical data are presented as number (percentage). The category ‘ACEIs/ARBs’ includes patients treated with either an ACE inhibitor or an ARB.

Abbreviations: ACEIs, Angiotensin‐converting enzyme inhibitors; AKI, acute kidney injury; ARBs, Angiotensin II receptor blockers. BMI, body mass index; BUN, blood urea nitrogen; CRRT, continuous renal replacement therapy; HB, hemoglobin; HCT, hematocrit; LAC, lactate; Scr, serum creatinine; MAP, mean arterial pressure; PLT, platelets; SOFA, Sequential Organ Failure Assessment; SPO_2_, pulse oxygen saturation; WBC, white blood cells.

a aggregates several specific groups with smaller sample sizes, including Asian, Asian Indian, Black/African, Black/African American, Black/Cape Verdean, Black/Caribbean Island, Hispanic or Latino (e.g., Salvadoran, Puerto Rican), Multiple Race/Ethnicity, Other, and Unknown.

Model 1 was unadjusted.

Model 2 adjusted for demographic and basic clinical factors: age, sex, insurance status, marital status, race, and BMI.

Model 3 further adjusted for hospitalization and vital sign factors: length of ICU stay, heart rate, mean arterial pressure (MAP), respiration rate, temperature, and SpO2.

Model 4 further adjusted for laboratory parameters and common ICU sedatives: glucose, white blood cell count, hemoglobin, hematocrit, platelet count, blood urea nitrogen, serum creatinine, lactate, and use of dexmedetomidine, midazolam, and propofol.

Model 5, our most fully adjusted model, additionally adjusted for key comorbidities, severity scores, and admission diagnosis: myocardial infarction, congestive heart failure, cerebrovascular disease, dementia, chronic pulmonary disease, renal disease, severe liver disease, sepsis, septic shock, Charlson Comorbidity Index, SOFA score, and the primary cause of ICU admission.

By presenting results from Model 1 through Model 5, we demonstrate the robustness of the association between ACEI/ARB use and reduced delirium risk, even after comprehensive adjustment for the observed baseline imbalances and a wide array of potential confounders.

### 2.5 Outcomes

The primary outcome under investigation was the incidence of delirium in patients who had been administered ACEIs or ARBs prior to their admission to the ICU. Secondary outcomes encompassed several key clinical endpoints. These included the duration of the patient's stay within the ICU, the frequency of acute renal events, the necessity for continuous renal replacement therapy (CRRT), and the utilization of vasopressors.

### 2.6 Delirium Assessment and Data Collection

Delirium was assessed daily throughout the ICU stay using the Confusion Assessment Method for the ICU (CAM‐ICU). Assessments were conducted during periods of minimal sedation, preferably during spontaneous awakening trials or when the Richmond Agitation‐Sedation Scale (RASS) score was ≥ −3. For patients who remained deeply sedated (RASS < −3), comatose, or otherwise unassessable for more than 24 consecutive hours, delirium status was recorded as ‘unassessable’ for that period, and these days were excluded from the primary delirium analysis. This approach is consistent with recommended practices in critical care delirium research to reduce misclassification bias. In line with standard ICU nursing practice, these assessments were documented approximately every 4 to 8 h, coinciding with nursing shift changes, to capture the fluctuating course of delirium. The first valid assessment occurred within 24 h of ICU admission. This high‐frequency, protocolized assessment schedule, intrinsic to the source database, maximizes sensitivity for detecting delirium episodes and is the most likely explanation for the relatively high cumulative incidence observed in our study.

### 2.7 Statistical Analysis

Categorical data were presented in the form of numerical counts along with their corresponding percentages. For continuous data, depending on the data distribution characteristics, they were presented either as the arithmetic mean with its associated standard deviation or as the median accompanied by the interquartile range. To evaluate the differences among continuous variables, a set of statistical analyses were carried out. Specifically, analysis of variance and rank ‐ sum tests were employed. To compare the characteristics of the study population across different outcome groups, the chi ‐ square test or Fisher's exact test was applied.

Due to 5% of vital signs and laboratory parameters being missing, the median values were used to substitute the missing data. In the case of height and weight, since the proportion of missing data was relatively low, ranging from 0.5% to 8%, no imputation method was adopted. To evaluate the outcomes related to delirium, survival curves were constructed through the application of the Kaplan–Meier approach, followed by the implementation of log‐rank analysis. A multivariate Cox regression analysis was carried out to explore the specific relationship between the incidence of delirium and the utilization of ACEIs or ARBs. To account for various covariates within the models, an adjusted Cox model was utilized. In total, five distinct models were employed during the regression analyses. Subsequent analyses that adjusted for relevant covariates incorporated subgroup analysis and interaction analysis. All statistical analyses were performed using STATA software (version 17.0), R packages (accessible via http://www.R-project.org, The R Foundation), and Free Statistics Software (version 1.8) [29]. In the process of Cox regression and model building, multiple imputation techniques were utilized to handle the missing values. Statistical significance was determined by a two‐tailed test, with a significance level defined as a *p*‐value less than 0.05.

## 3 Results

### 3.1 Participants

**Figure 1:**
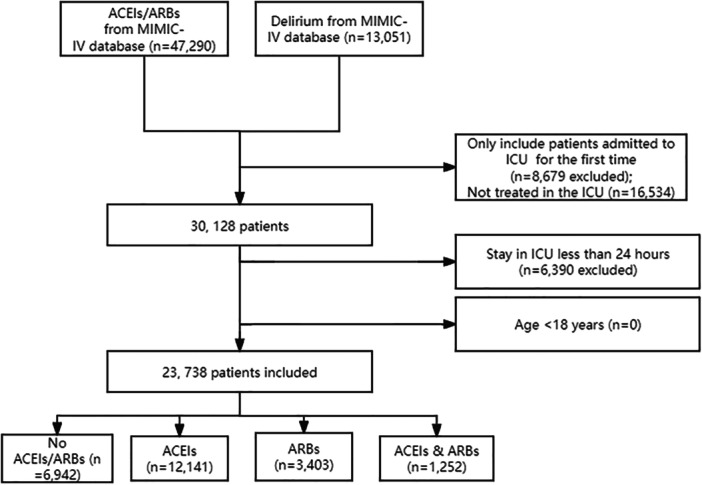
Flowchart of the selection of study participants.

In total, 66,239 patients satisfied the inclusion criteria. After the exclusion of patients with repeated admissions to the ICU, individuals younger than 18 years of age, those who persisted in using ACEIs or ARBs subsequent to their admission to the ICU, and patients with an ICU stay of less than 24 h, the final study cohort was composed of 15,666 patients. The process of selecting the study participants is presented in Figure 1.

### 3.2 Baseline Characteristics

The present study encompassed 15,666 patients, having an average age of 66.2 ± 15.5 years. Among these patients, 56.4% were male. Table 1 provides a comprehensive account of the baseline characteristics of the patient cohort. The distribution of ACEI/ARB use and delirium incidence, stratified by admission cause and age, is presented in Table 2. Delirium incidence varied significantly across admission categories in both patients aged ≥ 50 years (*p *< 0.001) and < 50 years (*p *= 0.016). A consistent descriptive pattern was observed: delirium rates were highest in admissions for drug poisoning/adverse reactions and lowest in those for cardiovascular diseases, across all medication groups (ACEIs, ARBs, or ACEIs/ARBs) and both age strata. The distribution of primary ICU admission causes differed significantly between these age strata (*p *< 0.001) (Supplementary Table S1).

**Table 2:** The prevalence of ACEI/ARB prescription and the incidence of delirium within each cause‐of‐admission category.

Variables	Total (*n* = 23,738)	Others (*n* = 10,134)	Trauma (*n* = 3,951)	Cardiovascular‐related diseases (*n* = 8,429)	Drug poisoning or adverse drug reactions (*n* = 1,224)	*p*
**Group (age ≥ 50 years)**						
ACEIs, *n* (delirium%)	10,865 (29.59)	4,482 (29.63)	1,315 (46.54)	4,731 (21.52)	337 (65.86)	< 0.001
ARBs, *n* (delirium%)	3,221 (30.92)	1,261 (29.66)	453 (48.12)	1,383 (20.17)	124 (63.71)	
ACEIs and/or ARBs, *n* (delirium%)	1,098 (34.70)	409 (31.30)	116 (47.41)	539 (31.17)	34 (76.47)	
No ACEIs/ARBs, *n* (delirium%)	5,437 (44.37)	2,565 (34.62)	1,315 (51.30)	1,085 (33.87)	472 (80.29)	
**Group (age < 50 years)**						
ACEIs, *n* (delirium%)	1,233 (28.14)	592 (29.73)	145 (46.21)	442 (15.16)	54 (62.96)	0.016
ARBs, *n* (delirium%)	169 (27.22)	83 (31.33)	20 (35)	55 (16.36)	11 (27.27)	
ACEIs and/or ARBs, *n* (delirium%)	151 (34.44)	55 (32.73)	12 (25)	75 (33.33)	9 (66.67)	
No ACEIs/ARBs, *n* (delirium%)	1,564 (39.78)	687 (35.79)	575 (47.46)	119 (39.17)	183 (73.56)	

*Note:* The category ‘ACEIs and/or ARBs’ encompassing both concomitant use and sequential switching.

Abbreviations: ACEIs, Angiotensin‐converting enzyme inhibitors; ARBs, Angiotensin II receptor blockers.

### 3.3 Associations of Delirium With ACEIs and ARBs

**Figure 2:**
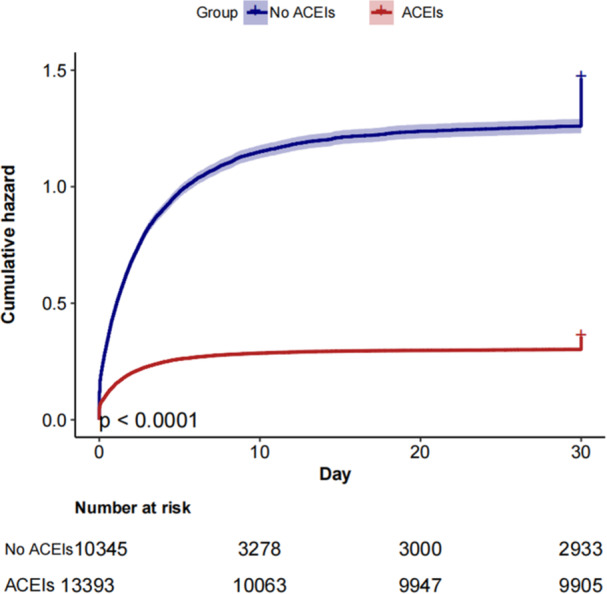
Cumulative incidence of delirium in critically ill patients who received ACEIs or ARBs and those who did not receive ACEIs/ARBs. This information was gathered over 30 days. Not adjusted.

Analysis via the Kaplan–Meier curve indicated a notable disparity in delirium rates during a 30‐day period between patients who did not receive angiotensin ‐ converting enzyme inhibitors (ACEIs) or angiotensin II receptor blockers (ARBs) and those who did. The log ‐ rank test showed a highly significant result (*p* < 0.0001), as depicted in Figures 2 and S1. The univariable associations between all baseline characteristics and the incidence of delirium are presented in Supplementary Table S3. Univariate analysis of the risk of delirium indicated that patients who received ACEIs or ARBs showed a significant association in delirium rates compared with those who not received [hazard ratio (HR) = 0.13, 95% confidence interval (95% CI): 0.12–0.14, *p* < 0.001] or ARBs (HR = 0.12, 95% CI: 0.11–0.14, *p* < 0.001) during the 30‐day period (Table S2). In the comprehensive multivariate Cox regression analysis (Table S2), the HRs associated with the use of both ACEIs and ARBs remained statistically significant across all the models considered. Specifically, the HRs fell within the range of 0.12 to 0.13, and in each case, the *p*‐values were less than 0.001. Following the adjustments for all covariates, a 87% lower risk of delirium was evident in patients who received ACEIs or ARBs (HR = 0.13, *p* < 0.001, Model 5).

### 3.4 Associations of Other Outcomes With ACEIs and ARBs

Upon the inclusion of all the covariates enumerated in Table 3, ACEIs and ARBs were associated with shortened ICU stays and ACEIs were associated with shortened vasoactive times (*p* < 0.05).

**Table 3:** Associations of the use of ACEs and/or ARBs with ICU stays and vasoactive times.

		Length of stay in the ICU (days)	Vasoactive time (hours)						
		Model 1	Model 2	Model 1	Model 2				
Variable	Total *N*	β (95% CI)	*p*	β (95% CI)	*p*	β (95% CI)	*p*	β (95% CI)	*p*
No ACEIs/ARBs	6,942	0(Ref)		0(Ref)		0(Ref)		0(Ref)	
ACEIs	12,141	−2.39 (−2.56 to −2.23)	< 0.001	0.05 (−0.09 to 0.19)	0.514	−28.41 (−30.92 to −25.9)	< 0.001	−2.12 (−4.28 to 0.05)	0.056
ARBs	3,403	−2.6 (−2.83to −2.37)	< 0.001	0.03 (−0.15 to 0.21)	0.746	−29.4 (−32.89 to −25.9)	< 0.001	−1 (−3.72 to 1.73)	0.473
ACEIs/ARBs	1,252	−2.48 (−2.82 to −2.15)	< 0.001	−0.03 (−0.26 to 0.21)	0.835	−27.94 (−33.07 to −22.81)	< 0.001	−2.71 (−6.38 to 0.95)	0.147
P for trend	23,738		< 0.001		0.905		< 0.001		0.315

*Note:* Model 1, Not adjusted; Mod**el 2**, adjusted for age, sex, insurance, marital status, race, BMI, ICU length of stay, heart rate, MAP, respiration rate, temperature, SPO_2_, glucose, WBC, HB, HCT, PLT, BUN, Scr, LAC, dexmedetomidine, midazolam, propofol, myocardial infarction, congestive heart failure, cerebrovascular disease, dementia, chronic pulmonary disease, renal disease, severe liver disease, sepsis, sepsis shock, Charlson Comorbidity Index, SOFA score.

Abbreviations: ACEIs, Angiotensin‐converting enzyme Inhibitors; ARBs, Angiotensin II receptor blockers; CI, Confidence interval.

A significant association was found between the use of ACEIs and ARBs and the risk of AKI on the 7th day. For ACEIs, the OR was 0.9, with a 95% CI ranging from 0.82 to 0.98. In the case of ARBs, the OR was 1.11, and the 95% CI was between 0.97 and 1.26. However, it is noteworthy that their use may potentially have association with a lower requirement for CRRT, with ORs ranging from 0.6 to 0.69 (Table 4).

**Table 4:** Associations of the use of ACEIs and/or ARBs with patients' AKIs and CRRTs.

		AKI							
		Model 1	CRRT	Model 2		Model 1	Model 2		
	Total *N*	OR (95% CI)	*p*	OR (95% CI)	*p*	OR (95% CI)	*p*	OR (95% CI)	*p*
No. ACEIs/ARBs	6,942	1(Ref)		1(Ref)		1(Ref)		1(Ref)	
ACEIs	12,141	0.74 (0.69–0.79)	< 0.001	1.25 (1.13–1.38)	< 0.001	0.28 (0.24–0.32)	< 0.001	0.78 (0.63–0.96)	0.019
ARBs	3,403	0.82 (0.74–0.89)	< 0.001	1.37 (1.21–1.55)	< 0.001	0.29 (0.23–0.36)	< 0.001	0.73 (0.54–0.99)	0.04
ACEIs/ARBs	1,252	0.94 (0.82–1.08)	0.376	1.47 (1.24–1.73)	< 0.001	0.46 (0.35–0.62)	< 0.001	0.98 (0.67–1.41)	0.894

*Note:* **Model 1**, Not adjusted; **Model 2**, adjusted for age, sex, insurance, marital status, race, BMI, ICU stay (number of days), heart rate, MAP, respiration rate, temperature, SPO_2_, glucose, WBC, HB, HCT, PLT, BUN, Scr, LAC, dexmedetomidine, midazolam, propofol, myocardial infarct, congestive heart failure, cerebrovascular disease, dementia, chronic pulmonary disease, renal disease, severe liver disease, sepsis, sepsis shock, Charlson Comorbidity Index, SOFA.

Abbreviations: ACEIs, angiotensin‐converting enzyme inhibitors; AKI, acute kidney injury; ARBs, angiotensin II receptor blockers; CI, confidence interval; CRRT, continuous renal replacement therapy; OR, odds ratio.

### 3.5 Subgroup and Sensitivity Analyses

**Figure 3:**
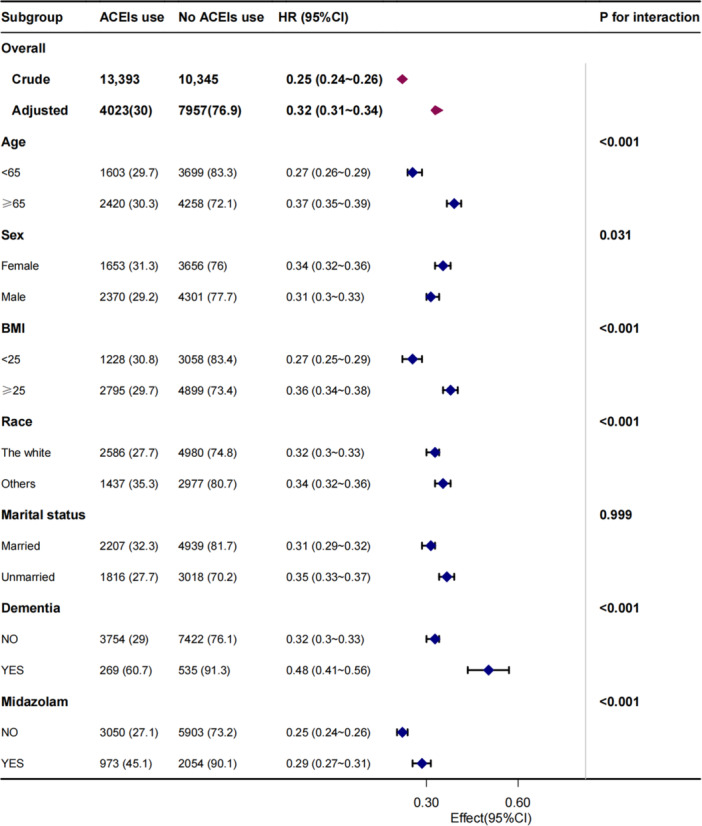
Associations of delirium in patients who received ACEIs or ARBs with those who did not receive ACEIs/ARBs, by baseline characteristics. Each stratification was adjusted for all factors excluding the stratified factor itself. Abbreviations: OR, odds ratio, BMI, body mass index; SOFA, Sequential Organ Failure Assessment.

The association of ACEIs and ARBs with lower delirium rate was more pronounced among individuals with the following characteristics: male, < 65 years old, unmarried, absence of dementia, and those who did not receive midazolam. No other significant interactions were observed within these subgroups (*p*‐value for interaction > 0.05) (Figures 3 and S2). A pre‐specified stratified analysis was performed by age group (< 50 vs. ≥ 50 years).

Adjustments of all covariates in the multivariate Cox regression model revealed HRs for delirium rate are 0.13 (95% CI: 0.13–0.15, *p* < 0.001) for the use of ACEIs and 0.13 (95% CI: 0.12–0.14, *p* < 0.001) for the use of ARBs (Table S2).

## 4 Discussion

The main outcome of this study reinforces the previously encouraging results regarding the application of ACEIs or ARBs for patients in critical condition. By utilizing a large ‐ scale database, our research produced solid evidence that supports the link between the use of ACEI or ARB and delirium in critically ill patients. Our results indicated a notable connection between the delivery of ACEIs or ARBs and the occurrence of delirium among patients who are critically ill. Moreover, the subgroup and sensitivity analyses we conducted enhance the reliability of these findings. This, in turn, indicates a statistical association between ACEI/ARB use and a reduced risk of delirium in critically ill patients based on observational data; however, this association does not establish causality, and prospective interventional studies are needed to confirm any potential protective effect.

To address confounding by indication, we performed a stratified analysis by age group. As expected, both ACEI/ARB use and delirium incidence were strongly associated with the primary admission diagnosis (e.g., highest ACEI/ARB use and lowest delirium in cardiovascular admissions; the opposite in drug poisoning). Critically, the inverse association between ACEI/ARB exposure and lower delirium risk maintained a consistent direction across major admission categories within both age strata. This internal consistency argues against confounding by indication as the sole explanation for our primary finding and lends support to a potential direct, protective biological effect of these medications.

### 4.1 Associations of Delirium With the Use of ACEIs or ARBs for Critically Ill Patients

The utilization of ACEIs or ARBs has been associated with a diminished incidence of delirium among critically ill patients. A study examining the preoperative use of ACEIs or ARBs in 539 patients who underwent surgical procedures for pulmonary hypertension demonstrated a decreased risk of postoperative delirium [30]. However, not all of the findings aligned with our conclusions [31, 32, 33]. A secondary analysis of data sourced from two simultaneous RCTs revealed that, after accounting for variables such as age, gender, race, comorbid conditions, and insurance status, the pre ‐ admission use of ACEIs or ARBs in the ICU was not associated with the occurrence of delirium [31]. Furthermore, another study's results indicated that the preoperative use of angiotensin system inhibitors were not related to a decrease in postoperative delirium [33]. Therefore, rigorous clinical investigations are imperative for a comprehensive assessment of the preventive and therapeutic efficacy of ACEIs and ARBs in delirium management.

This finding suggests an inverse association between ACEI/ARB use and the incidence of delirium in this critically ill cohort, which is possibly attributable to novel insights into the classical RAAS. These proposed mechanisms are based on preclinical data. Specifically, angiotensin II exerts neurotoxic effects via angiotensin type 1 receptors. Conversely, the RAAS demonstrates neuroprotective properties that are mediated by angiotensin (1–7), angiotensin III, and angiotensin IV [34]. ACEIs elevate brain substance P levels that are typically degraded by angiotensin‐converting enzymes [35], which, in turn, enhances the activity of the amyloid β‐degrading enzyme renin [36]. Furthermore, ACEIs facilitate the production of angiotensin (1–7). This peptide exhibits neuroprotective, anti ‐ inflammatory, and cerebral vasodilatory characteristics [34]. The adverse effects of angiotensin II on the brain are mainly exerted through its interaction with angiotensin receptor subtype 1 A. These effects include inflammation, hypertension, increased oxidative stress, impairment of the blood‐brain barrier, and neurotoxicity. Additionally, angiotensin II can activate angiotensin receptor II, thereby promoting nitric oxide production, neurite outgrowth, and brain development [37].

### 4.2 Associations of Other Outcomes With the Use of ACEIs or ARBs for Critically Ill Patients

In our investigation, we observed a significant association of ACEIs and ARBs with the risk of AKI on the 7th day. Nevertheless, these drugs have the potential to mitigate the likelihood of requiring CRRT. Although these findings may seem paradoxical, the increase in patients' creatinine levels could be attributed to the pharmacological vasodilatory effect on both renal afferent and efferent arterioles [38, 39]. Moreover, the inhibition of the RAAS may have an impact on outcomes not solely through hemodynamic and glomerular actions, but also by means of anti‐inflammatory or antithrombotic mechanisms [40]. Hence, a strong association of AKI induced by ACEIs or ARBs and adverse outcomes remains ambiguous.

The influence of the previous use of ACEIs or ARBs on the incidence of AKI in this patient group remains a subject of debate. A comparable study discovered a notable correlation between the use of ACEIs or ARBs and a more severe stage of sepsis‐related kidney injury during the hospital stay. Additionally, it reported an elevated incidence of stage 3 AKI within the initial week. However, there were no significant differences in any patient‐centered outcomes in this study, including the need for RRT or death [41]. A recent post‐hoc analysis conducted by Demiselle et al. [42] indicated that patients who had previously undergone RAAS inhibition were less likely to develop AKI during their ICU stay. Moreover, these patients might have derived benefits from a higher mean arterial pressure. Consequently, it remains to be determined through further research whether ACEIs or ARBs increase the risk of AKI and make CRRT necessary.

## 5 Strengths and Limitations

Our study possesses four primary advantages. Initially, we utilized a reliable and publicly available database, which in turn guaranteed the credibility and comprehensiveness of our data set. Secondly, our findings suggest that the use of ACEIs or ARBs leads to a substantial decrease in the risk of delirium among critically ill patients. Thirdly, a series of sensitivity analyses were carried out to verify the reliability of our results. (1) multiple ‐ model adjustments were applied in Cox regression analyses to control for confounding factors. The results demonstrated stability even after these adjustments, indicating the robustness of our regression models. (2) the categorizations of ACEIs or ARBs according to administration patterns and timing produced consistent results. This implies that the classification methods we adopted were appropriate and did not introduce significant biases. This comprehensive and rigorous analytical strategy strengthens the credibility and internal validity of our study findings, making them more reliable and trustworthy within the context of the research. Finally, because critically ill patients often have comorbid chronic heart and kidney disease [43], the widespread use of ACEIs or ARBs in these patients may be appropriate; thus our results have broader implications.

The limitations of our study are consistent with those typically found in observational research. Initially, an inherent constraint arises from the retrospective nature of our investigation. Unaccounted for potential confounding variables may exist, and our dataset lacks additional markers of inflammation beyond WBC counts. Second, when extrapolating the findings of our study, it is essential to exercise caution. This is due to the fact that the study was restricted to a single country (the United States of America) and a particular ICU environment. Even though our study was advantaged by a large and reasonably representative sample size, subsequent multicenter prospective studies could be conducted to validate our results. Third, a multitude of factors have been reported to influence the delirium risk associated with critically ill patients. These factors encompass educational level, smoking and alcohol consumption history, baseline cardiac function, hormone therapy, and other medical histories. However, this information was not incorporated into the MIMIC ‐ IV database. Additionally, our findings may be influenced by selection bias related to ICU admission etiology. For instance, younger patients—who are less frequently prescribed ACEIs/ARBs—are more likely to be admitted for acute conditions such as sepsis, trauma, or substance‑related complications, which themselves carry a higher baseline risk of delirium and could confound the observed association. Nevertheless, a sensitivity analysis adjusting for primary ICU admission diagnoses yielded results consistent with our main findings. Fourth, our retrospective clinical investigation did not elucidate the specific impact of ACEIs or ARBs on individual patients because of the lack of details regarding the duration of their administration. Fifth, evaluations of medication exposure prior to hospitalization or ICU admission are limited to prescribed medications; information about the dosage and duration of treatment are not available; nor is information available about whether prescriptions were filled or whether participants adhered to these medications, as prescribed. Sixth, ICU delirium is a syndrome influenced by many factors, including lighting, sleep, ICU environment, family relationships, and personalities. It is very difficult to reverse ICU delirium with a single drug, which is a limitation that warrants acknowledgment. Seventh, although we followed a standardized protocol to assess delirium during periods of minimal sedation, the possibility of under‐detection due to sustained deep sedation or neuromuscular blockade cannot be entirely ruled out, which may have influenced the observed incidence. Eighth, a key limitation of this study stems from the MIMIC‑IV modules used, which lack structured fields to identify treatment‑limitation orders such as Do‑Not‑Attempt‑Resuscitation (DNAR). This prevented direct adjustment for a potential source of confounding, as such orders likely reflect illness severity and care preferences that could influence both treatment and outcomes. While a sensitivity analysis excluding patients with ICU stays < 24 h was performed to indirectly address this concern, this remains an imperfect proxy. Our findings should therefore be interpreted with this limitation in mind.

Therefore, while our data suggest an association between preadmission ACEI/ARB use and a reduced risk of ICU delirium, this finding should be interpreted as hypothesis‐generating. Future prospective studies are required to clarify the role and timing of these medications before any clinical prioritization can be considered.

## Author Contributions

**Zhe Li:** investigation, conceptualization, methodology, validation, writing – original draft. **Shuo Liu:** validation, visualization, writing – original draft. **Qihai Wan:** writing – review and editing, visualization. **Yahui Niu:** funding acquisition, writing – review and editing. **Yi Yu:** methodology, data curation, supervision, project administration, writing – review and editing, writing – original draft.

## Ethics Statement

The research studies that included human participants underwent a comprehensive review and received approval from The Massachusetts Institute of Technology and Beth Israel Deaconess Medical Center. The legal guardians or next ‐ of ‐ kin of the participants furnished written informed consent for the individuals to take part in this study.

## Conflicts of Interest

The authors declare no conflicts of interest.

## Transparency Statement

The [Zhe Li, Shuo Liu, Qihai Wan, Yahui Niu, Yu Yi] affirms that this manuscript is an honest, accurate, and transparent account of the study being reported; that no important aspects of the study have been omitted; and that any discrepancies from the study as planned (and, if relevant, registered) have been explained.

## Supporting information


**Figure S1:** Cumulative incidence of delirium in critically ill patients who received ACEIs or ARBs and those who did not receive ACEIs/ARBs. This information was gathered over 30 days. Not adjusted.

**Figure S2:** Associations of delirium in patients who received ACEIs or ARBs with those who did not receive ACEIs/ARBs, by baseline characteristics. Each stratification was adjusted for all factors excluding the stratified factor itself. Abbreviations: OR, odds ratio, BMI, body mass index; SOFA, Sequential Organ Failure Assessment.

**Table S1:** Primary Causes of ICU Admission, categorized by age group.

## Data Availability

The data that support the findings of this study are available on request from the corresponding author. The data are not publicly available due to privacy or ethical restrictions.
