# Supplementary figures and images for: Impact of Prior Angiotensin‐Converting Enzyme Inhibitor and Angiotensin II Receptor Blocker Use on Delirium Incidence in ICU Patients: A Retrospective Study
Source: Health Sci Rep. 2026 Jun 22;9(6):e72676. doi: 10.1002/hsr2.72676 (PMC13285174; doi:10.1002/hsr2.72676)

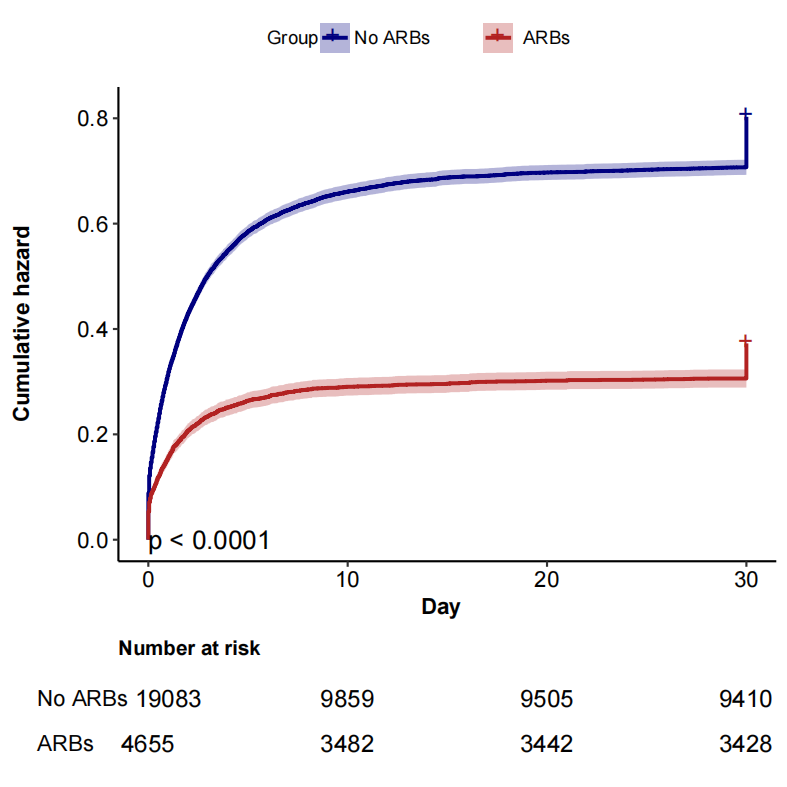

Supplement: Supplementary file 1 — Figure S1: Cumulative incidence of delirium in critically ill patients who received ACEIs or ARBs and those who did not receive ACEIs/ARBs. This information was gathered over 30 days. Not adjusted. [file HSR2-9-e72676-s003.tif]

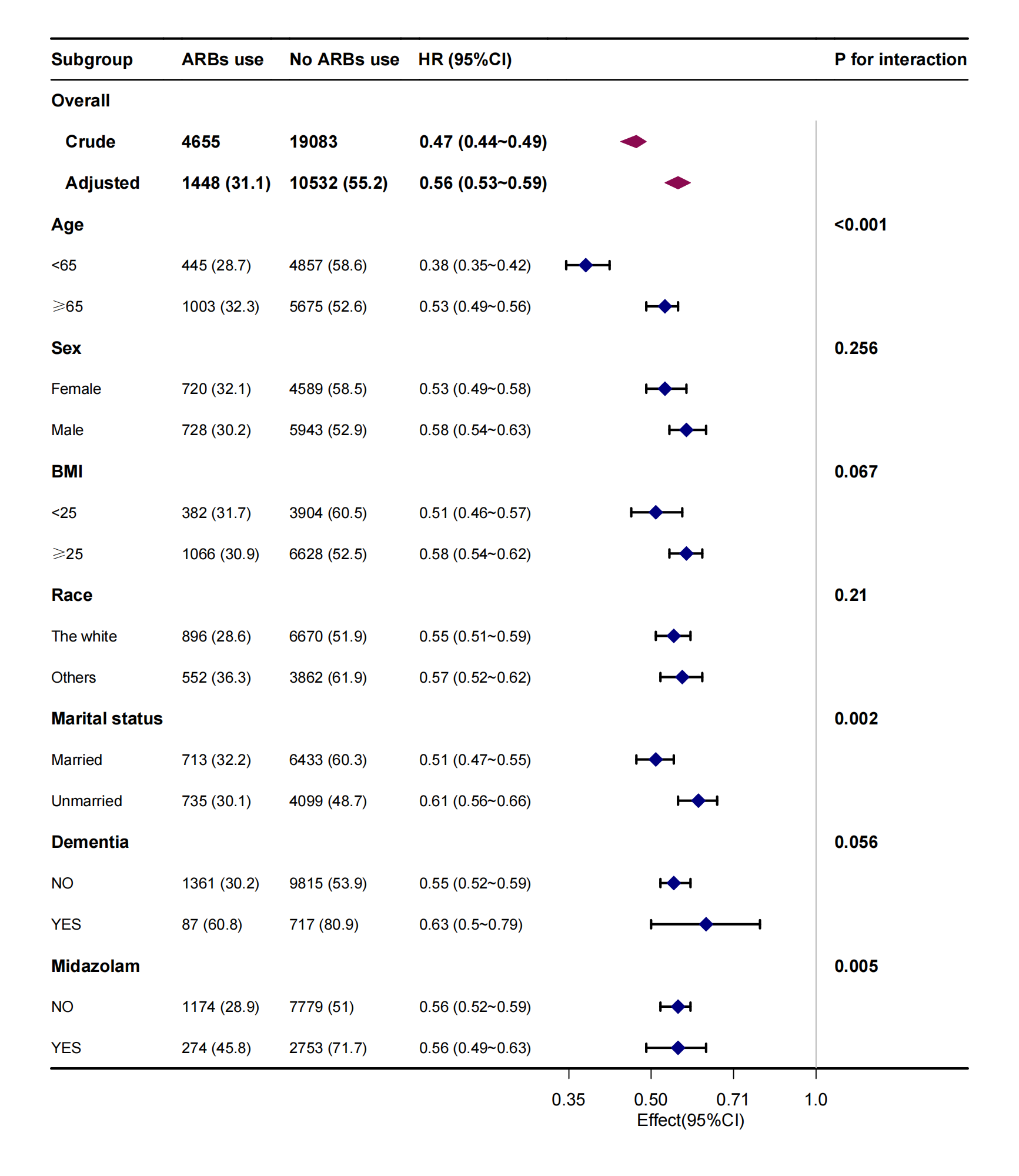

Supplement: Supplementary file 2 — Figure S2: Associations of delirium in patients who received ACEIs or ARBs with those who did not receive ACEIs/ARBs, by baseline characteristics. Each stratification was adjusted for all factors excluding the stratified factor itself. Abbreviations: OR, odds ratio, BMI, body mass index; SOFA, Sequential Organ Failure Assessment. [file HSR2-9-e72676-s001.tif]
