# Supplementary material for: Impact of Prior Angiotensin‐Converting Enzyme Inhibitor and Angiotensin II Receptor Blocker Use on Delirium Incidence in ICU Patients: A Retrospective Study
Source: Health Sci Rep. 2026 Jun 22;9(6):e72676. doi: 10.1002/hsr2.72676 (PMC13285174; doi:10.1002/hsr2.72676)
Supplement: Supplementary file 3 — Table S1: Primary Causes of ICU Admission, categorized by age group. [file HSR2-9-e72676-s002.docx]

**Supplementary Table S1:** Primary Causes of ICU Admission, categorized by age group.

| **Variables** | **Total (n = 23,738)** | **<50 (n = 3,038, mean ± SD: 42 ± 8.3years)** | **≥50 (n = 20,700, mean ± SD: 70 ± 11.1years)** | **p** |
| --- | --- | --- | --- | --- |
| **Diseases, n (%)** |  |  |  | < 0.001 |
| **Trauma** | 3,787 (16.1) | 645 (21.4) | 3,142 (15.3) |  |
| **Cardiovascular-related diseases** | 8,429 (35.8) | 691 (23) | 7,738 (37.6) |  |
| **Drug poisoning or adverse drug reactions** | 1,224 ( 5.2) | 257 (8.5) | 967 (4.7) |  |
| **Others** | 10,134 (43.0) | 1,417 (47.1) | 8,717 (42.4) |  |

**Supplementary Table S2:** Associations of ACEIs and/or ARBs with delirium.

|  | **Model 1** | **Model 2** | **Model 3** | **Model 4** | **Model 5** |
| --- | --- | --- | --- | --- | --- |
| **Variable** | **HR (95%CI)** | **HR (95%CI)** | **HR (95%CI)** | **HR (95%CI)** | **HR (95%CI)** |
| No ACEIs/ARBs | 1(Ref) | | | | |
| ACEIs | 0.13 (0.12-0.14) | 0.13 (0.12-0.14) | 0.12 (0.12-0.13) | 0.13 (0.12-0.14) | 0.13 (0.13-0.15) |
| ARBs | 0.12 (0.11-0.14) | 0.12 (0.11-0.14) | 0.12 (0.11-0.13) | 0.12 (0.11-0.13) | 0.13 (0.12-0.14) |
| ACEIs/ARBs | 0.13 (0.12-0.13) | 0.13 (0.12-0.13) | 0.12 (0.12-0.13) | 0.13 (0.12-0.13) | 0.13 (0.13-0.15) |

**Model 1:** Not adjusted

**Model 2: Model 1** adjusted for age, sex, insurance, marital status, race, and BMI

**Model 3: Model 2** adjusted for length of ICU stay (days), heart rate, MAP, respiration rate, temperature, and SPO_2_

**Model 4**: **Model 3** adjusted for glucose, WBC, HB, HCT, PLT, BUN, Scr, LAC, dexmedetomidine, midazolam, and propofol

**Model 5**: **Model 4** adjusted for myocardial infarction, congestive heart failure, cerebrovascular disease, dementia, chronic pulmonary disease renal disease, severe liver disease, sepsis, sepsis shock, Charlson Comorbidity Index, SOFA, Primary Causes of ICU Admission.

**Abbreviations:** /, or; ACEIs, Angiotensin-converting enzyme inhibitors; ARBs, Angiotensin II receptor blockers; HR, Hazard ratio; CI, Confidence interval. The category ‘ACEIs/ARBs’ includes patients treated with either an ACE inhibitor or an ARB.

**Supplementary Table S3**: Univariable associations between baseline characteristics and delirium incidence.

| **Variables** | **Total (n = 23,738)** | **Non-delirium (n = 11,822)** | **Delirium (n = 11,916)** | **p** |
| --- | --- | --- | --- | --- |
| **Age, n (%)** |  |  |  | < 0.001 |
| ≥ 65 | 9,806 (41.3) | 4,555 | 5,251 (44.1) |  |
| <65 | 13932 (58.7) | 7,267 | 6,665 (55.9) |  |
| **Sex, n (%)** |  |  |  | < 0.001 |
| Male | 10,096 (42.5) | 4,809 | 5,287 (44.4) |  |
| Female | 13642 (57.5) | 7,013 | 6,629 (55.6) |  |
| **BMI, n (%)** |  |  |  | < 0.001 |
| ≥25 | 7,652 (32.2) | 3,397 | 4,255 (35.7) |  |
| <25 | 16,086 (67.8) | 8,425 | 7,661 (64.3) |  |
| **Race, n (%)** |  |  |  | < 0.001 |
| White | 15,948 (67.2) | 8,458 | 7,490 (62.9) |  |
| Others | 7,790 (32.8) | 3,364 | 4,426 (37.1) |  |
| **Insurance, n (%)** |  |  |  | < 0.001 |
| Medicaid | 1,398 ( 5.9) | 589 | 809 (6.8) |  |
| Medicare | 11,681 (49.2) | 5,877 | 5,804 (48.7) |  |
| Others | 10,659 (44.9) | 5,356 | 5,303 (44.5) |  |
| **Marital status, n (%)** |  |  |  | < 0.001 |
| Married | 12,865 (54.2) | 5,756 | 7,109 (59.7) |  |
| Others | 10,873 (45.8) | 6,066 | 4,807 (40.3) |  |
| **Lactate, n (%)** |  |  |  | < 0.001 |
| ≥4 | 21,904 (92.3) | 11,388 | 10,516 (88.3) |  |
| <4 | 1,834 ( 7.7) | 434 | 1,400 (11.7) |  |
| **Sofa, n (%)** |  |  |  | < 0.001 |
| ≥6 | 12,523 (52.8) | 7,366 | 5,157 (43.3) |  |
| <6 | 11,215 (47.2) | 4,456 | 6,759 (56.7) |  |
| **Myocardial infarct, n (%)** |  |  |  | < 0.001 |
| No | 19,141 (80.6) | 9,086 | 10,055 (84.4) |  |
| Yes | 4,597 (19.4) | 2,736 | 1,861 (15.6) |  |
| **Congestive heart failure, n (%)** |  |  |  | < 0.001 |
| No | 16,616 (70.0) | 7,935 | 8,681 (72.9) |  |
| Yes | 7,122 (30.0) | 3,887 | 3,235 (27.1) |  |
| **Cerebrovascular disease, n (%)** |  |  |  | < 0.001 |
| No | 19,670 (82.9) | 10,064 | 9,606 (80.6) |  |
| Yes | 4,068 (17.1) | 1,758 | 2,310 (19.4) |  |
| **Dementia, n (%)** |  |  |  | < 0.001 |
| No | 22,711 (95.7) | 11,594 | 11,117 (93.3) |  |
| Yes | 1,027 ( 4.3) | 228 | 799 (6.7) |  |
| **Chronic pulmonary disease, n (%)** |  |  |  | 0.042 |
| No | 17,983 (75.8) | 9,023 | 8,960 (75.2) |  |
| Yes | 5,755 (24.2) | 2,799 | 2,956 (24.8) |  |
| **Renal disease, n (%)** |  |  |  | 0.223 |
| No | 18,675 (78.7) | 9,339 | 9,336 (78.3) |  |
| Yes | 5,063 (21.3) | 2,483 | 2,580 (21.7) |  |
| **Severe liver disease, n (%)** |  |  |  | < 0.001 |
| No | 22,836 (96.2) | 11,695 | 11,141 (93.5) |  |
| Yes | 902 ( 3.8) | 127 | 775 (6.5) |  |
| **Sepsis, n (%)** |  |  |  | < 0.001 |
| No | 10,968 (46.2) | 6,844 | 4,124 (34.6) |  |
| Yes | 12,770 (53.8) | 4,978 | 7,792 (65.4) |  |
| **Sepsis shock, n (%)** |  |  |  | < 0.001 |
| No | 21,774 (91.7) | 11,581 | 10,193 (85.5) |  |
| Yes | 1,964 ( 8.3) | 241 | 1,723 (14.5) |  |
| **Diabetes, n (%)** |  |  |  | < 0.001 |
| No | 15,431 (65.0) | 7,233 | 8,198 (68.8) |  |
| Yes | 8,307 (35.0) | 4,589 | 3,718 (31.2) |  |
| **Dexmedetomidine, n (%)** |  |  |  | < 0.001 |
| No | 19,566 (82.4) | 10,964 | 8,602 (72.2) |  |
| Yes | 4,172 (17.6) | 858 | 3,314 (27.8) |  |
| **Midazolam, n (%)** |  |  |  | < 0.001 |
| No | 19,323 (81.4) | 10,412 | 8,911 (74.8) |  |
| Yes | 4,415 (18.6) | 1,410 | 3,005 (25.2) |  |
| **Propofol, n (%)** |  |  |  | < 0.001 |
| No | 12,696 (53.5) | 7,443 | 5,253 (44.1) |  |
| Yes | 11,042 (46.5) | 4,379 | 6,663 (55.9) |  |

Data are presented as n (%) unless otherwise specified. Percentages in the ‘Total’ column represent the proportion of the subgroup in the entire cohort. Percentages in the ‘Delirium’ column represent the incidence of delirium within that specific subgroup.
